# Trajectory of quality of life among patients with cancer at the end of life: a longitudinal survey study

**DOI:** 10.1007/s11136-025-03923-0

**Published:** 2025-02-18

**Authors:** Henriette Tind Hasse, Trine Kjær, Søren Rud Kristensen

**Affiliations:** Danish Centre for Health Economics, Department of Public Health, University of Southern Denmark, Campusvej 55, Odense, DK-5230 Denmark

**Keywords:** Health-related quality of life, End-of-life, Cancer, Cancer care, Healthcare, Health economics

## Abstract

**Purpose:**

Measuring and understanding the determinants of HRQoL is essential to the delivery of effective and high-quality end-of-life (EoL) care to patients with cancer. Despite this, the evidence base remains sparse and with much of the existing literature relying on data from cross-sectional studies and clinical trials.

**Aim:**

The objective of this study was to describe HRQoL in a population of patients with cancer leading up till death using both the generic preference-based scale European Quality of Life 5 Dimensions 5 Level Version and the disease-specific scale European Organization for the Research and Treatment of Cancer Quality of Life Questionnaire.

**Methods:**

Using a longitudinal prospective study design, HRQoL data was collected in four waves over the course of one year. The population consisted of all patients who received cancer-targeted drug treatment at the Department of Oncology at Odense University Hospital, Denmark. Only patients who died during the data collection period were included.

**Results:**

HRQoL in patients with cancer was stable for most months and close to the level of the general Danish population at the same age but deteriorated considerably in the last three months of life. The same pattern was observed for both HRQoL scales.

**Conclusion:**

Despite current efforts to deliver high-quality EoL care to patients with cancer, we see a general decrease in HRQoL in the months leading up to death. The generic and disease-specific HRQoL scales do capture different dimensions of HRQoL which also, by construct, are weighted differently in the two approaches.

## Introduction

Cancer is one of the leading causes of death and continues to impact the lives of millions worldwide profoundly. Historically, the primary focus of cancer treatment has been overall survival or progression-free survival [1]. However, health-related quality of life (HRQoL) is increasingly recognised as a pivotal outcome [2] particularly when cancer is beyond curative measures [3, 4].

Therefore, knowledge about HRQoL and its determinants in patients with cancer near the end of life (EoL) is important to health care providers and policymakers.

To measure HRQoL, various disease-specific and generic tools have been developed. Generic measures enable researchers to evaluate the impact of treatments on both health status and quality of life regardless of the disease type, treatment, or patient group [5]. One of the most widely used generic tools is the EQ-5D-5 L [6].

Condition-specific measures identify symptoms unique to a specific condition and the immediate effect of that condition on an individual’s quality of life [5]. The European Organization for the Research and Treatment of Cancer Quality of Life Questionnaire (EORTC-QLQ-C30) is a widely used cancer disease-specific HRQoL instrument [7]. As the tool is not preference-based and does not yield utility scores, it cannot be used directly in economic evaluations [8].

Despite the importance of HRQoL, evidence remains sparse and with much of the existing literature relying on HRQoL data measured by various scales and, in most cases, from cross-sectional studies and clinical trials [9–11] capturing one moment in time that may not be representative of the individual’s general HRQoL state [12].

Raijmakers et al. investigated a cohort of 892 diseased cancer patients who had replied to the EORTC-QLQ-C30 either 3, 3–6, 6–9, or 9–12 months before death. They found that patients who responded three months before death had a significantly lower HRQoL than individuals who answered earlier. Furthermore, they found that patients with cancer within their last year of life had a significantly lower HRQoL than the general population [13]. The study did not measure the development in HRQoL over time. Lee et al. [14] used the Functional Assessment of Cancer Therapy– General questionnaire to assess HRQoL in 345 patients with cancer every three months until death. Results showed a deterioration of HRQoL as death approached. Their conclusion emphasized the importance of systematic HRQoL monitoring, early identification, and referrals to palliative care to prevent the steep decline in HRQoL [14]. As with the study by Raijmakers et al., Lee et al. evaluated HRQoL using a disease-specific measurement tool. The individuals invited to participate in the study by Lee et al. were informed of their terminal state, which may in itself have influenced HRQoL [15]. Another cross-sectional study applied both EQ-5D-5 L and EORTC-QLQ-C30 to investigate HRQoL in patients with end-stage cancer. Both instruments were found to be applicable, but EQ-5D-5 L showed a pronounced ceiling effect with 13% reporting perfect health [11]. Strong ceiling (or floor) effects can make the instrument insensitive to change in the top and bottom of the scale [16, 17].

Randomized Controlled Trials (RCT) are another source of knowledge regarding HRQoL in cancer patients [9, 10]. The RCT population often comprises individuals with low-risk profiles who are younger and less co-morbid than the general cancer population [18]. Moreover, many RCTs only measure HRQoL during the intervention and not close to death [19]. Other studies have investigated HRQoL using real-world data focusing on specific cancer types only [20–23], or using caregivers’ HRQoL assessment as a surrogate measure [24, 25]. While some studies have found generic HRQoL measurement tools to be less sensitive to changes in HRQoL in patients with cancer than the disease-specific tools, others have found the opposite, making results inconclusive, especially at EoL [26–28].

In this study, our primary objective was to describe the development in HRQoL over time in patients with cancer near the end of life, using both the generic preference-based EQ-5D-5 L and the disease-specific EORTC-QLQ-C30 questionnaires. Our secondary objective was to show the trajectory of the subdomains of both HRQoL measures over time to show the drivers of the development in overall HRQoL.

## Data and methods

### Study design

This study was designed as a longitudinal prospective study. We included all patients suffering from a solid tumour cancer who had received cancer-targeted drugs within the last 90 days of entry at Odense University Hospital (OUH), Denmark. The entry date was 17 January 2022. No criteria regarding disease length, cancer type, or life expectancy were applied. In total, 1,522 patients met the inclusion criteria and were invited to participate. Only individuals who died during the data collection were included in this study (*N* = 256). Death and time of death was identified retrospectively after data collection was completed.

Using the unique Personal Identification Number (CPR number) assigned to all individuals with permanent Danish residency, data from the comprehensive and valid Danish administrative registers were collected for the entire study population and merged with the survey data and administrative hospital data.

### HRQoL survey

**Fig. 1:**
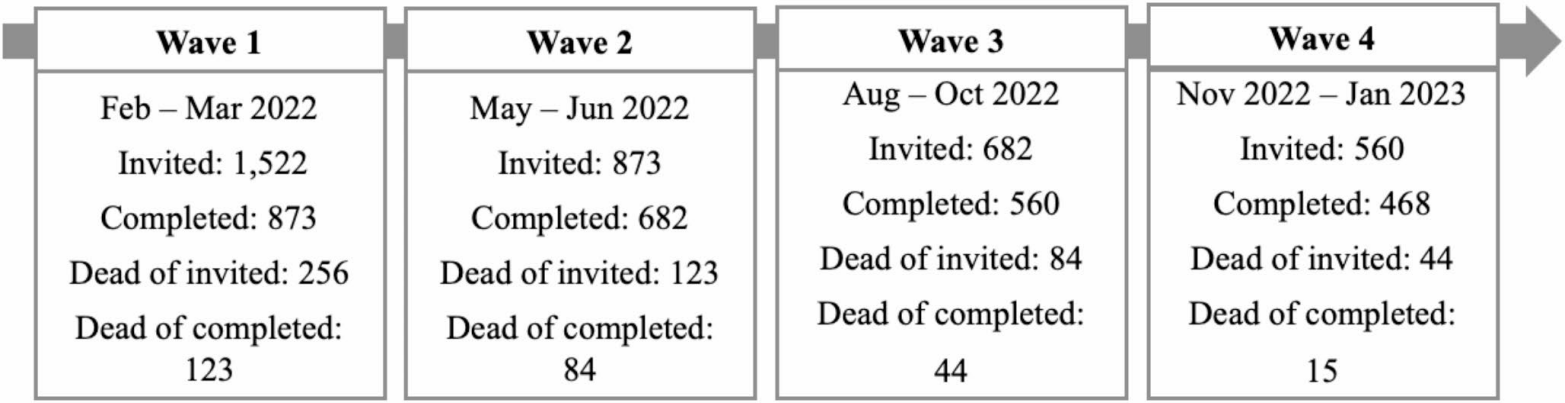
Distribution of survey incl. number of respondents^1^

Information on HRQoL was collected using an online survey distributed in four waves over the course of one year, using both the validated EQ-5D-5 L [29, 30] and EORTC-QLQ-C30 [31]. The first wave was distributed on 17 February 2022, followed by waves two, three and four every subsequent three months. Wave four was distributed on 24 November 2022. The surveys were open for approximately one month each, making the total data collection period approximately one year. For detailed information, see Fig. 1 and Appendix Table 1.

#### EQ-5D-5 L

The EQ-5D-5 L quality of life assessment questionnaire [29, 30] is a generic HRQoL measurement tool. The EQ-5D-5 L measures HRQoL on five dimensions: Mobility, Self-care, Daily activity, Pain and discomfort, and Anxiety and depression. The responses are on an ordinary scale and rank from 1 (no problem/pain/anxiety) to 5 (extreme problem/pain/anxiety) [29]. The five-digit score corresponds to a particular health state, representing the individual’s self-reported health condition. For example, a score of ‘11111’ signifies perfect health with no problems in any dimension, while ‘55555’ represents the worst health state with extreme problems in all dimensions. Each possible health state generated from the EQ-5D-5 L score is assigned a health state utility value. The Danish values applied in this study were derived from interviews with individuals representative of the general Danish adult population. The respondents each valued seven health states using discrete-choice experiment and 10 using the composite time trade-off [30]. The EQ-5D-5 L questionnaire also contained a VAS scale where the participant indicates their current health state on a scale from 0 (worst health imaginable) to 100 (best health imaginable) [29]. The VAS score was not included in the EQ-5D-5 L score and thus not presented in the current study.

#### EORTC-QLQ-C30

The EORTC-QLQ-C30 HRQoL questionnaire is a cancer-specific HRQoL measurement tool that comprises 30 items. The scales are as follows with number of items within the scales in (): global health status/QoL (2), physical functioning (5), role functioning (2), emotional functioning (4), cognitive functioning (2), social functioning (2), fatigue (3), nausea and vomiting (2), pain (2), dyspnoea (1), insomnia (1), loss of appetite (1), constipation (1), diarrhoea (1) and financial difficulties (1) [32]. A linear transformation to a ‘0-100’ ratio scale was carried out according to the EORTC-QLQ-C30 scoring manual. A high score for the global health status/QoL scale and functional scales represents a high/healthy level of QoL and functioning. Opposite, a high score for a symptom scale represents a high level of symptomatology/problems [33]. After The EORTC-QLQ-C30 sum score was calculated from the mean of 13 of the 15 QLQ-C30 scales (The global quality of life scale and financial impact scale was not included). Prior to calculating the mean, the symptom scales were reversed to obtain a uniform direction of all included scales [33, 34].

Eligible patients were invited via a secure email (e-Boks^2^) with a link to the survey. Only respondents were invited to participate in the subsequent questionnaire.

### Information from hospital administrative data and registers

From the hospital administrative data, information regarding treatment, type of cancer, age, sex, and time of death were obtained. Type of cancer was determined via the International Classification of Disease 10th version (ICD-10) DC00*-DC80* and grouped according to cancer site.

From the Danish Households registry, we obtained information on habitat status (living alone or not) and information regarding children. From the educational registry, we obtained information regarding the level of education, which was defined according to the International Standard Classification of Education (ISCED) system (low, medium, high). From the National Patient Register, time of diagnosis was collected and used to define time from diagnosis until death. For each respondent, we calculated the number of days from each survey response till date of death. We generated a new variable, ‘*Months from death*’, in which we classified the responses by months from death. This grouping enabled us to evaluate how HRQoL developed over time. All information from the Danish registries was collected at time of entry. Data from the registers and hospital administrative data was used to describe the population and was partially included in the investigation of the trajectory of HRQoL.

### Statistical analysis

#### Descriptive statistics

Descriptive statistics, including mean, standard deviation and p-values for group comparison, were applied to describe the demographic and clinical characteristics of both the respondents and non-respondents. Non-respondents were included to determine if the population sample was representative of the total population of interest. Groups were compared using Pearson’s chi-square test for categorical variables and t-test for continuous variables.

#### Trajectory of HRQoL over time

To show the trajectory of HRQoL over time as measured by both the EQ-5D-5 L and the EORTC-QLQ-C30 we employed a Generalized Estimating Equations (GEE) regression model with an exchangeable within group correlation structure which accommodated the panel data structure. To explore model adequacy and compare various distributional and link functions, we employed the Quasi-likelihood Information Criterion (QIC) statistics. For the EQ-5D-5 L (0–1 scale), we employed a Gaussian family and a log Link and for the EORTC-QLQ-C30 (0–100 scale), we employed a Poisson family with an Identity link. The regressions were performed both adjusted and unadjusted. In the adjusted analysis, age, sex, education, and time from diagnosis until death were included. The results were displayed as average marginal treatment effects at the mean value of each employed independent variable. Time was displayed in months from death.

#### Trajectories of HRQoL subdomains

To illuminate the drivers of the trajectories in the overall HRQoL measures, we estimated the development in each subdomain of both the EQ-5D-5 L and the EORTC-QLQ-C30 over time.

The EQ-5D-5 L subdomains (mobility, self-care, usual activities, pain/discomfort, and anxiety/depression) are all measured on a 1–5 ordinal scale and we therefore estimated a random effects ordered logistic model that took the panel data structure into account. We estimated separate models for each subdomain with dummy variables representing ‘*Months from death*’ as the independent variables. We then plotted the predicted marginal probabilities of each response on the ordinal scale at each time point.

For the Global Health score/QoL, functional, symptomatic, and item-specific scales that comprised the EORTC-QLQ-C30, we estimated the same GEE panel data model used for the overall EORTC-QLQ-C30 outcome with a Poisson family and Identity link. Again, we estimated the model for each subdomain score converted to the 0–100 ratio scale with dummies for ‘*Months from death*’ as the independent variables and plotted the predicted score at each time point.

The statistical analyses were conducted on a secure remote server hosted by Statistics Denmark using Stata 18 software (StataCorp. 2021. *Stata Statistical Software: Release 18.* College Station, TX: StataCorp LLC). The statistical level of significance was set at 5%.

### Ethics

This study was approved by the Research Ethics Committee (RECID275) and by the Research & Innovation Organization (RIO) at the University of Southern Denmark in accordance with the Data Protection Regulation under registration number 11.262. Permission to obtain information on patients regarding personal identification number (CPR number), disease status, and treatment was granted by the Region of Southern Denmark (journal no. 21/41200).

## Results

### Descriptives

Out of 1,522 invited participants, 256 died during the data collection period. Of these, 123 persons responded to at least the first survey wave (min) and 15 of these persons responded to all four surveys (max), with an average of 2.16 repeated responses per person. Respondents and non-respondents were statistically similar in terms of age, sex, having children, living alone, time from diagnosis to death, and education (p-values > 0.05). However, there were slight differences in the proportions of patients with gastroenterological, genitourinary, and lung cancers, with these types being slightly over-represented in the responder group. The mean time from the last response to death was 6 months, ranging from 3 days to 11.6 months (Table 1). Mean HRQoL values are shown in Table 2. For the distribution of responses by month prior to death, see Appendix Table 3. See Appendix Table 1 for descriptive statistics for the total invited population. In the total invited population, the respondents and non-respondents were statistically significantly different according to number of deaths, age, having children, living alone and level of education.

**Table 1:** Sample characteristics by respondence status. Percentages in parentheses unless stated otherwise

	Respondents	Non-respondents	*P* value
Population, n	123	133	
Mean age (SD)	68(9.20)	66(11.5)	0.096
Sex			0.719
*Female*	48(39)	54(41)	
Has children	107(87)	114(86)	0.986
Living alone	27(22)	28(21)	0.791
Education			0.429
*Low*	28(23)	41(31)	
*Medium*	62(50)	67(51)	
*High*	33(27)	24(18)	
Mean time from diagnosis until death (SD)	3.2 years (1.5)	3.1 years (1.2)	0.530
Mean time from last response until death (SD)	6 months (3)	-	-
Cancer type			0.730
*Gastroenterological incl. Colorectal*	42(34)	38(29)	
*Lung*	23(19)	28(21)	
*Genitourinary incl. Prostate*	19(15)	16(12)	
*Central Nervous System*	8(6)	13(10)	
*Breast*	9(7)	12(9)	
*Gynaecological*	9(7)	11(8)	
*Head and neck*	7(6)	4(3)	
*Malignant Melanoma*	5(4)	6(5)	
*Other*	2(2)	4(3)	

**Table 2:** Mean HRQoL sum score as measured by the EQ-5D-5 L and the EORTC-QLQ-C30. Standard deviation in ()

Scoring instrument	Mean HRQoL
Respondents (n)	123
HRQoL– EQ-5D-5 L (SD)	0.77(0.014)
HRQoL– EORTC-QLQ-C30 (SD)	71(1)
Number of observations	266

### Trajectory of HRQoL

**Fig. 2:**
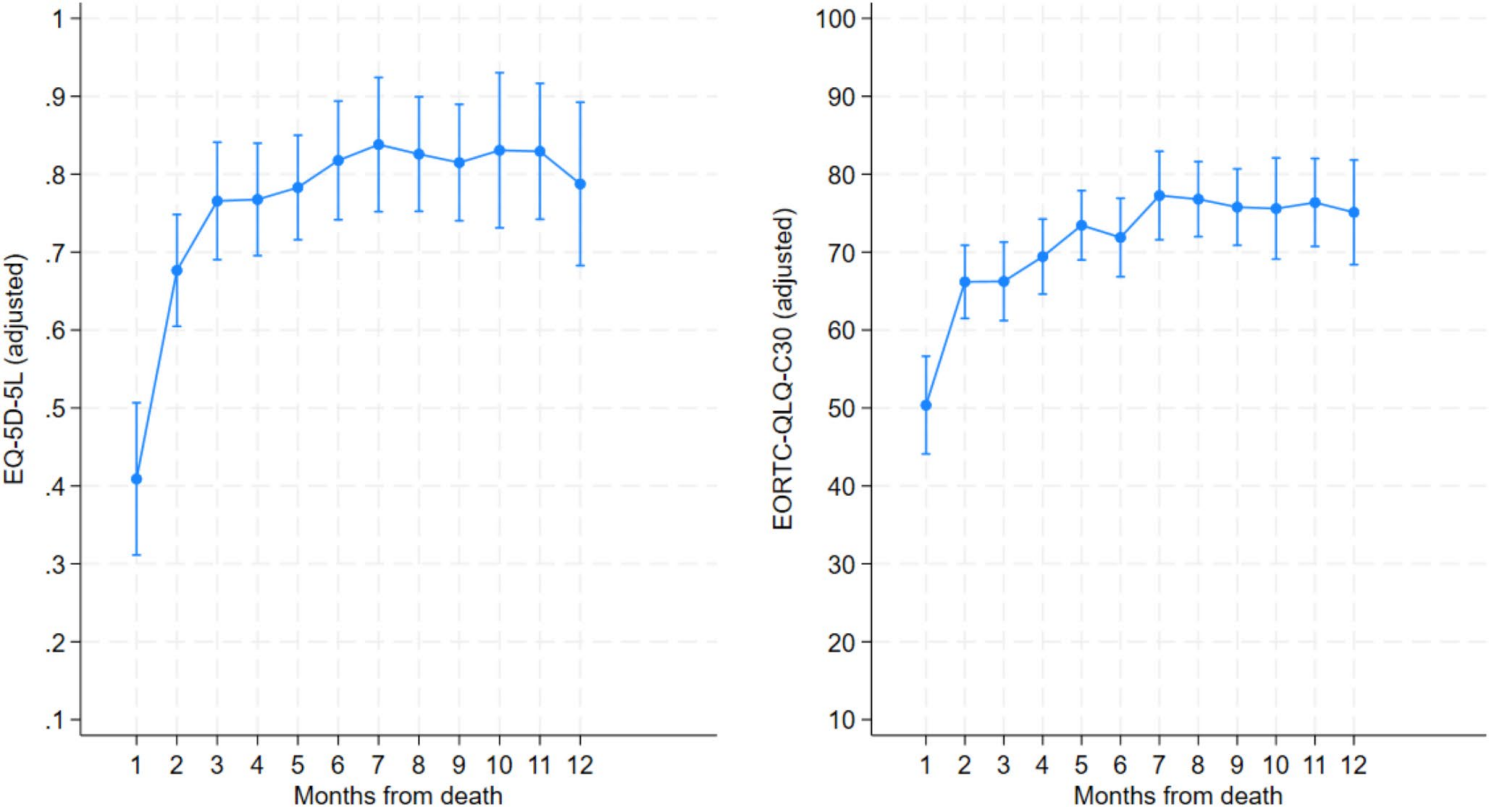
Mean EQ-5D-5 L and EORTC-QLQ-C30 HRQoL displayed by month before death. 95% confidence interval included

As presented in Fig. 2 HRQoL, measured using the EQ-5D-5 L and EORTC-QLQ-C30, deteriorated as time of death approached. A steeper decline from the second to the last month was seen for the EQ-5D-5 L compared with the EORTC-QLQ-C30. For the EQ-5D-5 L, the highest HRQoL score was 0.83 (95% CI 0.743 to 0.917) 11 months prior to death. The EQ-5D-5 L score was 0.41 (95% CI 0.312 to 0.507) in the last month before death. For the EORTC-QLQ-C30, the highest score was 78 (95% CI 74.30 to 81.02) 7 months prior to death. The score for the last month before death was 50.5 (95% CI 47.53 to 53.49). All scores in both analyses were statistically significant with a p-value of < 0.001. Only the time from diagnosis until death statistically significantly affected HRQoL as measured by the EORTC-QLQ-C30. Please see Table 3 and Appendix Table 4 for further details. Including age, sex, education, and time from diagnosis until death did not alter the results very much compared with the unadjusted analysis. Unadjusted results not displayed.

**Table 3:** Adjusted regression analysis displaying HRQoL over time as measured by the EQ-5D-5 L and the EORTC-QLQ-C30 incl. 95% confidence interval. Results displayed as margins

	EQ-5D-5 L	EORTC-QLQ-C30
Respondents (n)	123	123
Month from death		
1	0.409***	50.51***
	(0.312–0.507)	(47.53–53.49)
2	0.677***	66.13***
	(0.605–0.749)	(63.55–68.70)
3	0.766***	66.19***
	(0.691–0.842)	(63.42–68.95)
4	0.768***	69.49***
	(0.696–0.841)	(66.81–72.17)
5	0.784***	73.63***
	(0.716–0.851)	(71.06–76.20)
6	0.818***	71.25***
	(0.742–0.894)	(68.38–74.13)
7	0.839***	77.66***
	(0.753–0.925)	(74.30–81.02)
8	0.827***	76.73***
	(0.753–0.900)	(73.88–79.57)
9	0.815***	75.47***
	(0.741–0.890)	(72.61–78.33)
10	0.832***	75.75***
	(0.732–0.931)	(71.95–79.55)
11	0.830***	76.12***
	(0.743–0.917)	(72.82–79.43)
12	0.788***	75.74***
	(0.683–0.893)	(71.81–79.68)
Observations	266	266
ci in parentheses		
*** *p* < 0.01, ** *p* < 0.05, * *p* < 0.1		

### Trajectories of subdomains over time

#### EQ-5D-5 L

**Fig. 3:**
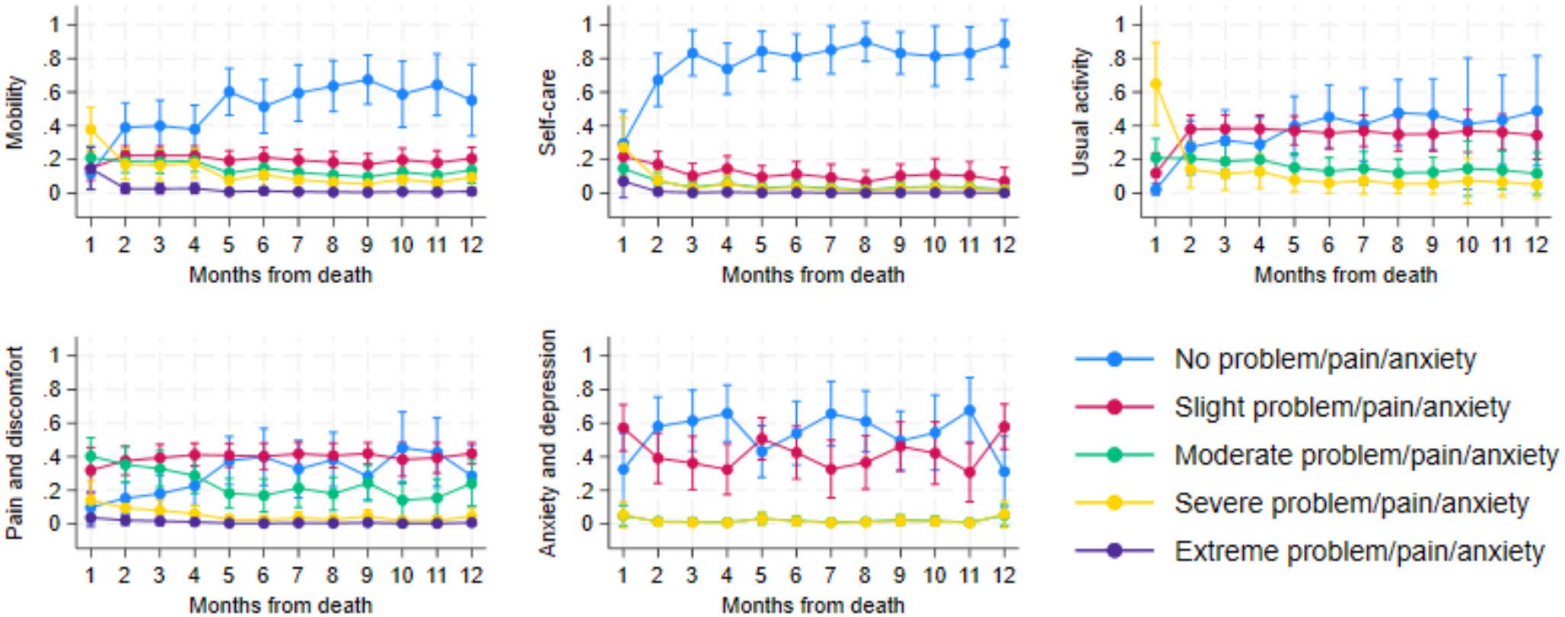
*Ordered logit of the EQ-5D-5 L functions displayed as odds of having outcomes ranging from ‘No problem/pain/anxiety’ to ‘Extreme problem/pain/anxiety’ by month before death. 95% confidence interval included*

In Fig. 3, the five dimensions of the EQ-5D-5 L are displayed. In each dimension, a higher score indicates worse HRQoL. In all dimensions, the probability of reporting *No problem/pain/anxiety* was lower when approaching the last month of life. In all dimensions, except for anxiety/depression, the risk of reporting *severe problem/pain/anxiety* increased as the end of life approached, especially for usual activity, self-care, and mobility. In the dimensions usual activity and anxiety/depression, only four outcomes were present (one through four), as no one responded *Extreme problem/pain/anxiety*.

The median EQ-5D-5Lvalues by month before death show that usual activity had the steepest rise in function loss within the last month of life and was affected from the 10th month prior to death. Mobility score increased from the fourth and the last month before death. Self-care was unaffected until the last month before death, which saw a sharp increase in severity. For more see Fig. 7 in the appendix.

#### EORTC-QLQ-C30 Global Health Score/QoL

The global health score/QoL is not included in the EORTC-QLQ-C30 sum score. The score was 65 (95% Confidence interval (CI) 39.24 to 49.02) 12 months prior to time of death and was reduced to 44.13 (95% CI 39.24 to 49.02) in the last month before death. All scores were statistically significantly different from the last month with a p-value of < 0.001. Please see Appendix Table 5.

#### EORTC-QLQ-C30 functioning scales

**Fig. 4:**
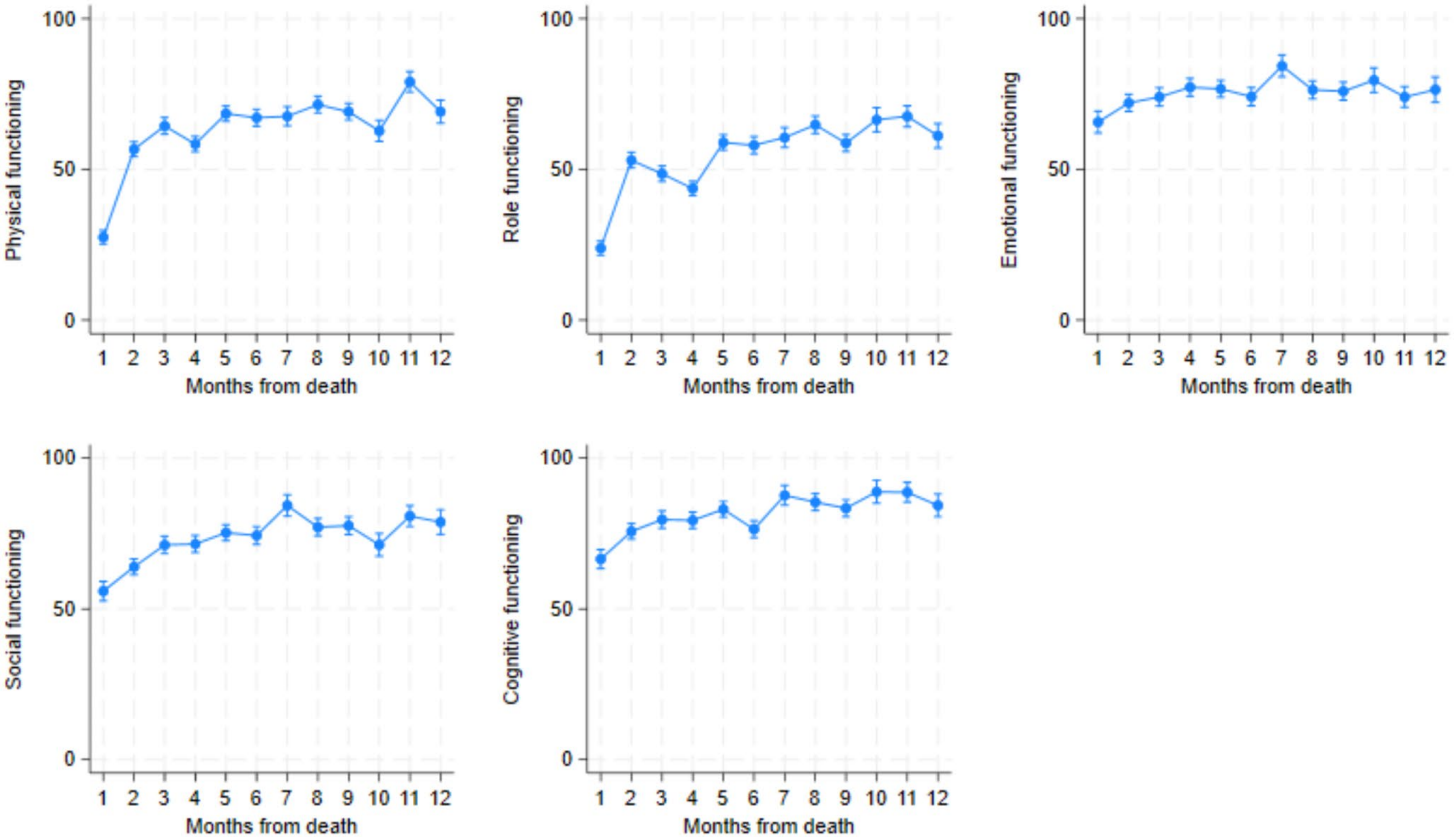
Mean EORTC-QLQ-C30 functioning scale scores displayed by month before death. 95% confidence intervals are included

The EORTC-QLQ-C30 functioning scales are presented in Fig. 4. For this scale, a higher score indicates less affected function, hence a less effected HRQoL. Physical and role functioning decreased sharply between the second and last month before death. Social functioning decreased from the third month before death. Cognitive functioning decreased in the last month before death. Emotional functioning declined slightly from the third month prior to death.

### EORTC-QLQ-C30 symptom scale

**Fig. 5:**
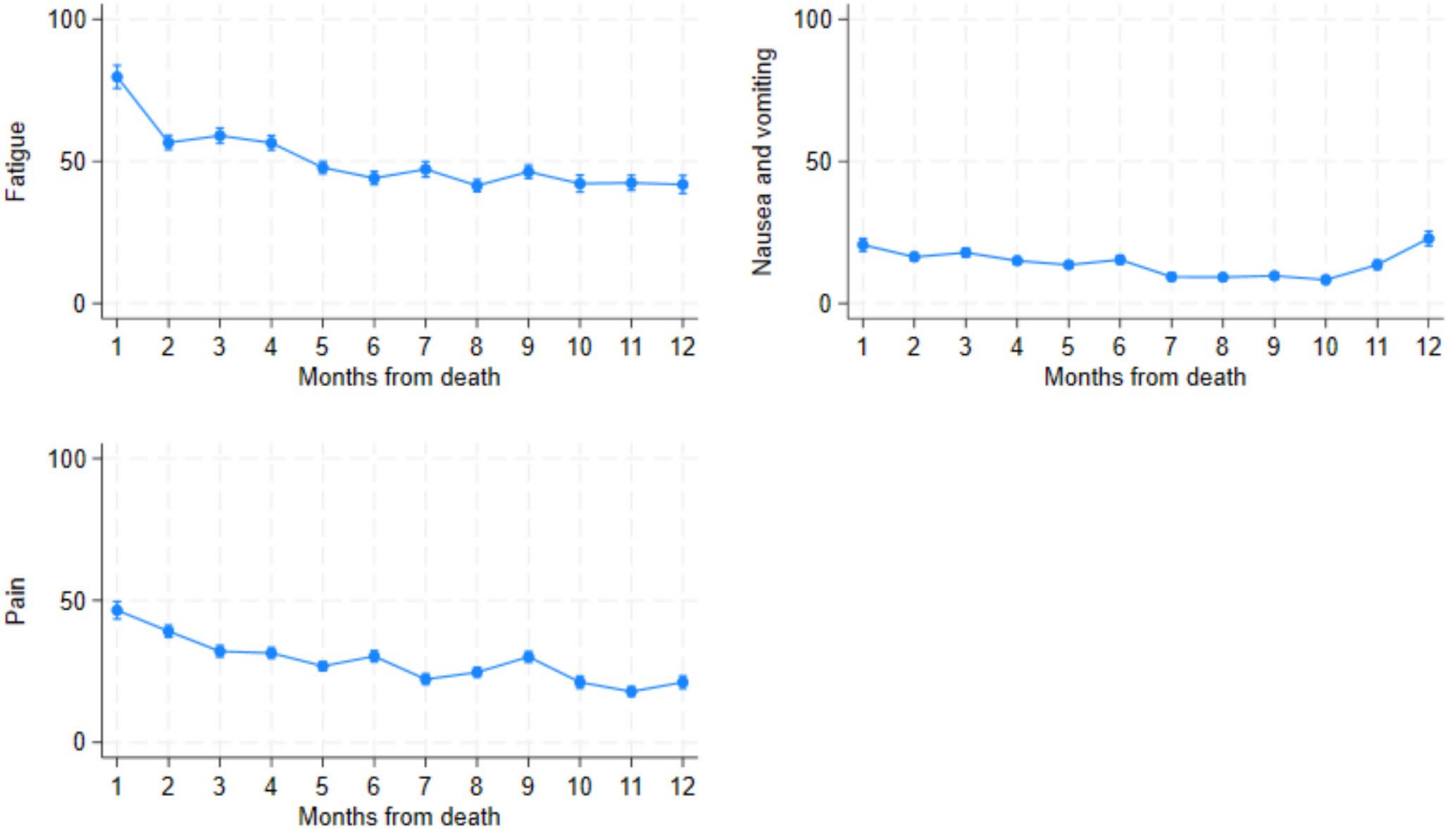
Mean EORTC-QLQ-C30 symptom scale scores displayed by month before death. 95% confidence intervals included

The EORTC-QLQ-C30 symptom scales are presented in Fig. 5. For the EORTC-QLQ-C30 symptom scale, a higher score indicates severer symptoms, thus affecting the HRQoL negatively. The symptom fatigue increased the most in the last months before death. Nausea and vomiting peaked 12 months prior to death. Pain worsened toward the last month before death with a somewhat steeper rise from the third month.

### EORTC-QLQ-C30 single items

**Fig. 6:**
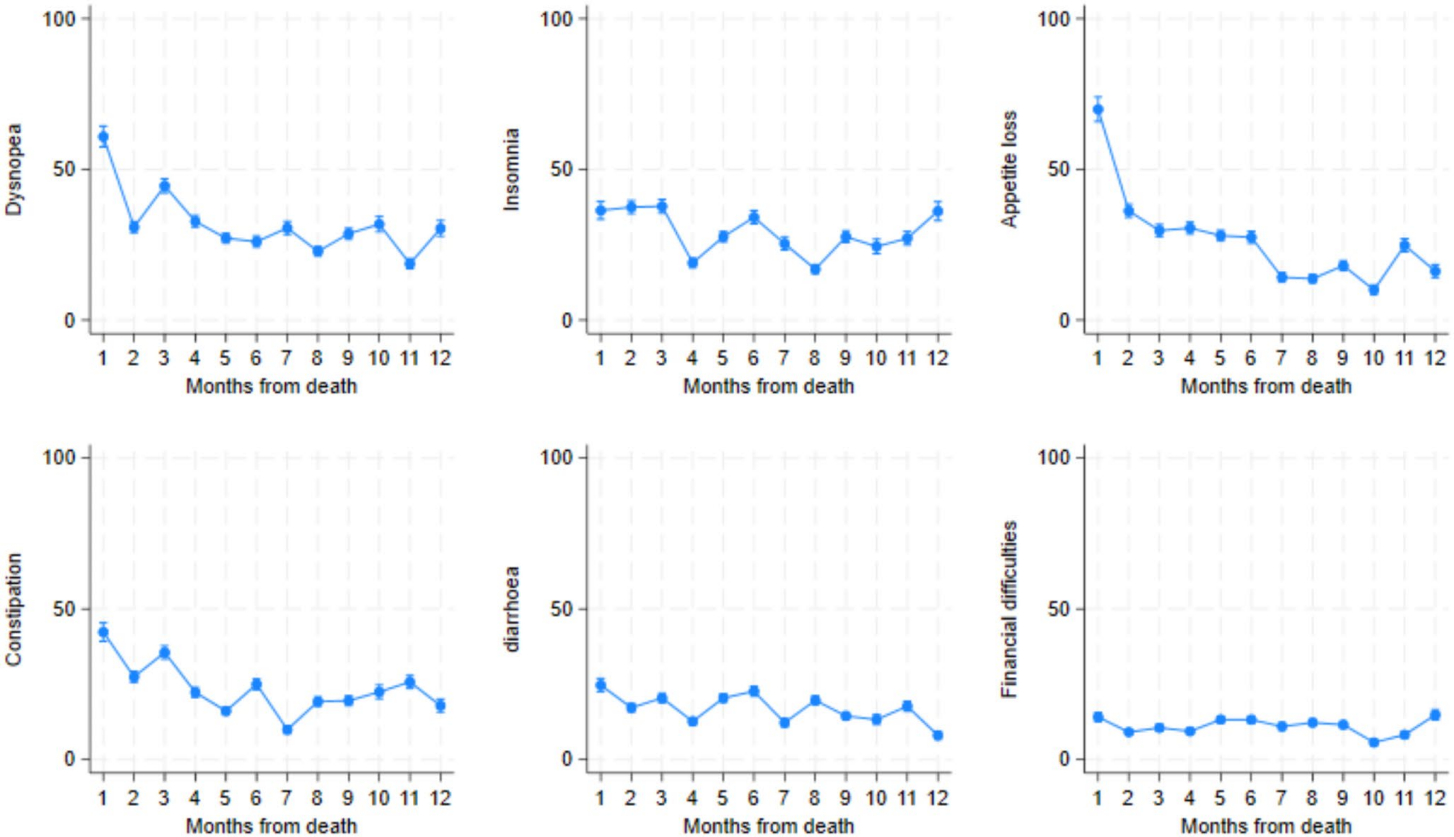
EORTC-QLQ-C30 single-item scale scores displayed by month before death. 95% confidence intervals included

The EORTC-QLQ-C30 single-item scales are presented in Fig. 6. In the single-item scale scores, a higher score indicates a more severe symptom and a more negatively affected HRQoL. Dyspnoea increased sharply in the last two months before death, whereas insomnia decreased from the third month before death. Loss of appetite rose sharply from the third month prior to death.

## Discussion

Out of the invited individuals that died during or right after data collection, nearly half agreed to participate and responded to the first questionnaire as a minimum. Both HRQoL, as measured by the EQ-5D-5 L and by the EORTC-QLQ-C30, indicated a decline as the time of death approached. The deconstructed EQ-5D-5 L showed that the decrease in HRQoL was especially affected by loss of mobility and usual activities. The deconstructed EORTC-QLQ-C30 showed that the decline was especially driven by a loss in physical, role, and cognitive functioning as well as an increase in severity of the symptoms fatigue, dyspnoea, and loss of appetite.

A strength in our study was the ability to link data from different sources via the patients’ unique CPR numbers, allowing us to enrich our prospective data with retrospective registry data without asking for extra information from the patients. We were able to compare the respondents with non-respondents using hospital administrative data and Danish registry data. Our results showed that the respondents and non-respondents were comparable in many aspects, e.g. according to age, referral to SPC, and length of disease trajectory. This is important, as a major challenge in collecting survey data is that a select group tends to respond when invited to participate in surveys [35].

In a newly conducted study by Jensen et al. [36], the authors found an average EQ-5D-5 L score of 0.89 for the age group 60–69 in the Danish general population. This is similar to the mean EQ-5D-5 L score of 0.84 the 11th month before death found in this study. When approaching the time of death, especially in the last six months, HRQoL deteriorates, ending at a score of 0.44. This implies that when measured by the EQ-5D-5 L, HRQoL in patients with cancer is comparable to the general population until approximately the last six months of life. In the EORTC-QLQ-C30, the global health status/QoL measured at 12 months prior to death was 65, which was 8 points below the Danish population average as found by Juul et al. in 2014 [4]. While our results indicate a decline, they also highlight that HRQoL is not stationary, making continued measures over time important. A cross-sectional study would have concluded very different results depending on the timing of the study.

Several previous studies have found evidence that patients suffering from incurable cancer wish to prioritize the improvement or stabilization of their HRQoL [1, 14, 37]. This entails that the healthcare professionals know what factors are influenced by the disease and how to alleviate them. If the goal of care at EoL is improving HRQoL, continuous screening of HRQoL could help detect deterioration and thereby begin to remedy HRQoL sooner. Our results indicate that especially the ability to be mobile, perform self-care, and usual activities affect HRQoL. These are skills that are related to a feeling of autonomy and ‘being normal’ [38]. Houska and Loučka find that emphasis of care should be placed on supporting the patients in their daily activities and contribution to others [38]. This aligns with our finding that Role functioning in the EORTC-QLQ-C30 measurement was a driver of low HRQoL, indicating the importance of being able to fulfil one’s role, which may be given inadequate attention in a healthcare setting. Which type of role functioning the patient finds most important is individual and may vary according to age and sex, among other things [39]. Moreover, we found that physical functioning and loss of appetite worsened towards the time of death. Both of these have previously been identified as prognostic indicators in patients with cancer [40]. This suggests that applying these measurements continuously when caring for patients with end-stage cancer could help detect when patients are nearing the time of death, thereby securing timely initiation of appropriate EoL care. This could include termination of active cancer targeted treatment, referral to SPC, ensuring that the patient is able to spend more time at home (if possible), initiating extended pain management or other symptom relief. This may enable the patient to better fulfil a role or preform an activity that they find meaningful. More research is needed to investigate the relationship between HRQoL decline and time of death. As we only include cancer decedents and evaluate data retrospectively, we cannot causally claim that continuous measuring HRQoL would improve prospective accuracy of identifying EoL decline.

Knowledge about the functions and abilities that effect HRQoL at EoL may aid healthcare professionals in initiating correct intervention aimed at alleviating the steep decline at EoL. Of course, a complete alleviation is not realistic, as a decline in HRQoL at EoL is to be expected, despite care quality.

In this study, we applied both the EQ-5D-5 L and the EORTC-QLQ-C30 to the same population. This allowed us to evaluate HRQoL in a more nuanced manner compared to applying a single tool. The two tools are different in construction and designed to measure different elements of the disease, but as our results indicate, both tools capture the decline in HRQoL and the sum scores of both instruments follow a similar curve when patients approach the time of death. Our results do not indicate an insensitivity in the EQ-5D-5 L; on the contrary, it seems that the preference-based weighted categories capture change in HRQoL well. Ceiling and floor effects are recognized as a problem when using the EQ-5D-3 L [41], but this problem with the EQ-5D-5 L is not evident in our results, which is in line with previous studies that highlight ceiling effects as primarily being a problem in healthy populations [42].

Depending on the research question, a strategic approach might involve utilizing a combination of tools, an approach suggested by others [43, 44]. The EORTC-QLQ-C30 holds valuable data for customizing EoL care, specifically in addressing prevalent symptoms associated with cancer treatment. Meanwhile, the EQ-5D-5 L offers crucial insights that should be considered in inevitable priority discussions, as the EQ-5D-5 L outcome is comparable across different diseases and interventions. As cancer has a distinct EOL trajectory [45] and future research could focus on collecting equivalent data from patients at EoL suffering from diagnosis other than cancer.

Despite the population size being within range of previous literature [37, 46, 47], the number of participants prevents us from incorporating adequate independent variables in the models, thus risking residual confounding. Moreover, this study may be subject to selection bias as only responders are invited to participate in following questionnaires. This approach was chosen in an attempt to not pressure a vulnerable population excessively.

Future research could try to include a larger sample, to enable more independent variables or matching methods. Moreover, applications of the forthcoming Danish EORTC-QLQ-C30 utility weights could further enhance the comparison of the instruments. To compare EQ-5D-5 L and EORTC-QLQ-C30 more adequately, further studies including state-of-the-art mapping techniques are needed to verify the results of the current study. Mapping predicts health state utility values when no preference-based measure is included in a study. It estimates the relationship between non-preference-based and generic preference-based measures using statistical associations, requiring overlap in their descriptive systems and administration to the same population. Brazier et al. [48] reviewed various models used for this, such as OLS, generalized linear models, tobit, CLAD, 2-part models, and response mapping. Studies assess model performance using metrics like predicted mean and standard deviation, median, range, AIC, BIC, R², pseudo-R², RMSE, MSE, and MAE.

### Conclusion

This study contributes important new insights into the measurement of the trajectory of HRQoL for patients with cancer at EoL. Despite efforts to deliver high-quality EoL care to cancer patients, we see a general and pronounced decrease in HRQoL in the months leading up to death. While a decrease is to be expected, continuous measurement of HRQoL may help capture decline in a timely manner and thereby enable actions aimed at improving HRQoL or slowing the negative development. Whereas the results are very similar across aggregated scales, the generic and disease-specific HRQoL scales do capture different dimensions of HRQoL. The EQ-5D-5 L enables a comparison of HRQoL across diseases, and thus can be used as input for priority setting, but the EORTC-QLQ-C30 provides a more nuanced picture of HRQoL in patients with cancer, which is valuable in the effort to deliver tailored and effective care. Therefore, choosing an instrument for measuring HRQoL in patients with incurable cancer requires consideration.

## Appendix

**Appendix Table 1:** Population characteristics by respondence status. Percentages in parentheses unless stated otherwise

	Respondents	Non-respondents	*P*-value
Population, n *	826	630	
Died	124 (15.0%)	132 (21.0%)	0.03
Mean age (SD)	65.404 (11.420)	62.922 (13.674)	< 0.001
Sex			0.883
*Female*	469 (56.9%)	361 (57.3%)	
Has children	724 (87.9%)	529 (84.1%)	0.039
Living alone	171 (20.8%)	163 (25.9%)	0.021
Education			< 0.001
*Low*	579(70.1%)	448(71.1%)	
*Medium*	186 (22.5%)	107 (17.0%)	
*High*	39 (4.7%)	33 (5.2%)	
*Missing*	22 (2.7%)	42 (6.7%)	
Cancer type			0.141
*Gastroenterological incl. Colorectal*	177(21.4%)	110(17.5%)	
*Lung*	100 (12.1%)	81 (12.9%)	
*Genitourinary incl. Prostate*	126(15.2)	100(15.9)	
*Central Nervous System*	28 (3.4%)	33 (5.2%)	
*Breast*	198 (24.0%)	177 (28.1%)	
*Gynaecological*	80 (9.7%)	48 (7.6%)	
*Head and neck*	49 (5.9%)	25 (4.0%)	
*Malignant Melanoma*	53 (6.4%)	45 (7.1%)	
*Other*	15(1.8%)	11(1.7%)	

*Of the 1522 invited, 66 did not have available register data

**Appendix Table 2:** Sample size and rate across survey waves

Wave 1	Response
Completed	123 (48%)
Start without completion	12 (5%)
Non-response	105 (40%)
“Do not want to participate”	18 (7%)
Total	258 (100%)
Wave 2	
Completed	84 (68%)
Start without completion	1 (1%)
Non-response	25(20%)
“Do not want to participate”	3 (2%)
Died between distribution	10 (9%)
Total	123
Wave 3	
Completed	44 (52%)
Non-response	9 (11%)
“Do not want to participate”	2 (2%)
Died between distribution	29 (35%)
Total	84
Wave 4	
Completed	15 (35%)
Start without completion	1 (2%)
Non-response	27 (61%)
“Do not want to participate”	1 (2%)
Total	44

**Appendix Table 3:** Number of responses per month from death

Month from death	Frequency	Percent
Respondents (n)	123	-
1	15	5.63
2	24	9.02
3	25	9.40
4	29	10.90
5	32	12.03
6	26	9.77
7	19	7.14
8	26	9.77
9	26	9.77
10	14	5.26
11	18	6.76
12	12	4.45
Total	266	100

**Appendix Table 4:** Adjusted regression analysis displaying HRQoL over time as measured by the EQ-5D-5 L and the EORTC-QLQ-C30 incl. 95% confidence interval. Results displayed as coefficients

	EQ-5D-5 L	EORTC-QLQ-C30
Respondents (n)	123	123
Month from death (ref. month 1)		
2	0.505***	14.63***
	(0.249–0.762)	(10.85–18.41)
3	0.631***	14.68***
	(0.378–0.883)	(10.82–18.54)
4	0.637***	19.29***
	(0.395–0.879)	(15.77–22.80)
5	0.645***	22.10***
	(0.401–0.889)	(18.50–25.71)
6	0.696***	19.99***
	(0.443–0.950)	(15.97–24.01)
7	0.720***	27.64***
	(0.470–0.970)	(23.56–31.72)
8	0.717***	26.22***
	(0.472–0.962)	(22.50–29.94)
9	0.687***	24.25***
	(0.435–0.939)	(20.29–28.20)
10	0.734***	25.66***
	(0.477–0.991)	(21.20–30.12)
11	0.719***	25.76***
	(0.467–0.972)	(21.66–29.86)
12	0.680***	24.93***
	(0.410–0.950)	(20.13–29.74)
Age	0.000137	0.0628
	(-0.00464–0.00492)	(-0.0929–0.218)
Female (ref. Male)	-0.0688	-1.290
	(-0.158–0.0208)	(-4.223–1.644)
Level of education (ref. low)		
Medium	0.0140	1.600
	(-0.0910–0.119)	(-1.906–5.106)
High	-0.0440	-2.178
	(-0.162–0.0735)	(-6.010–1.654)
Time from diagnosis to death	2.24e-06	-0.00276***
	(-5.84e-05–6.29e-05)	(-0.00483– -0.000701)
Constant	-0.878***	50.49***
	(-1.293– -0.463)	(38.84–62.15)
Observations	266	266
ci in parentheses		

*** *p* < 0.01, ** *p* < 0.05, * *p* < 0.1

**Fig. 7:**
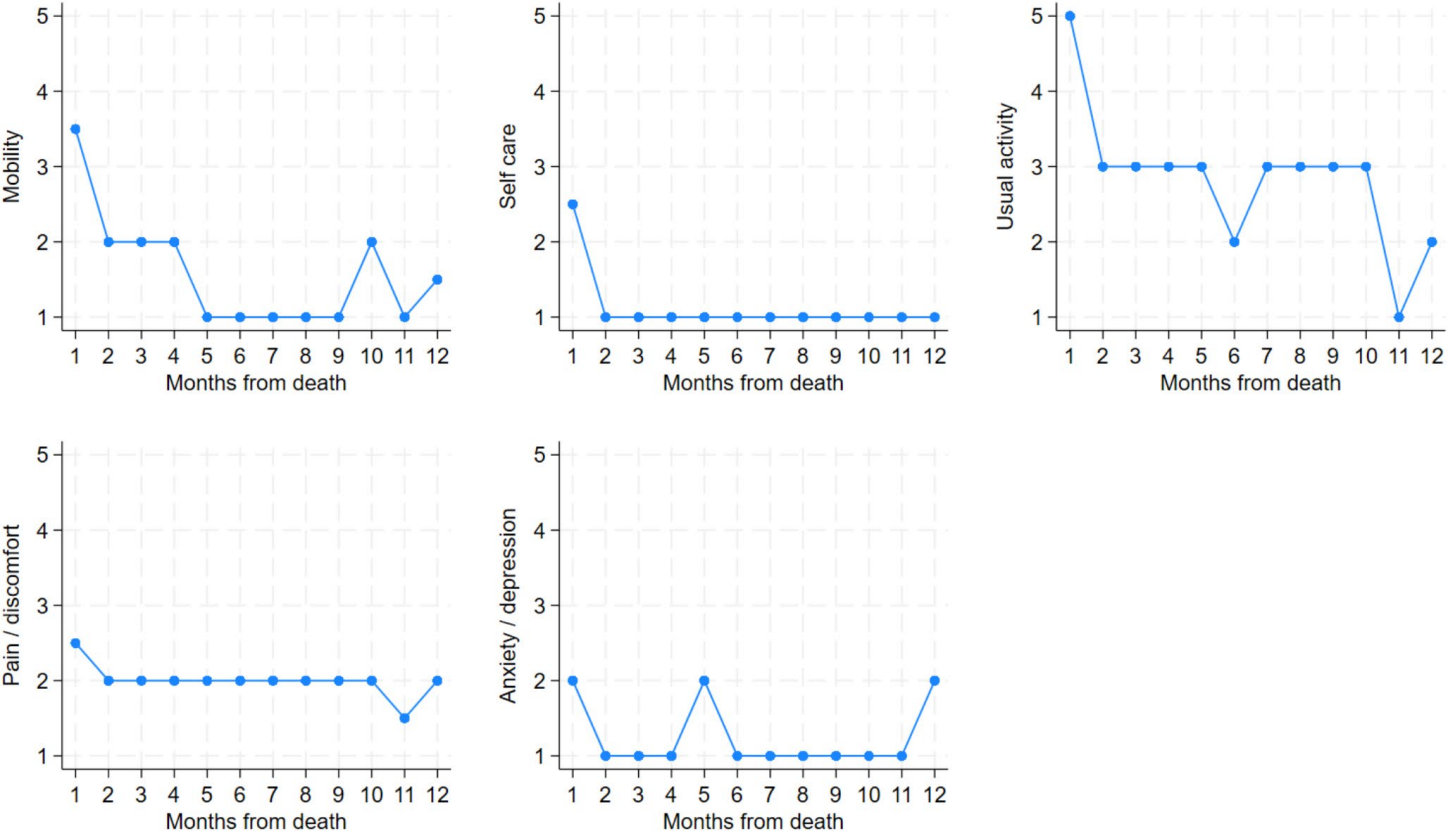
EQ-5D-5 L deconstructed dimensions presented as median values by month before death

**Appendix Table 5:** Global Health score/QoL by month from death. 95%CI included

	Global health status/QoL
Respondents (n)	123
Month from death	
1	44.13***
	(39.24–49.02)
2	46.02***
	(41.81–50.23)
3	47.92***
	(44.32–51.51)
4	49.81***
	(46.73–52.89)
5	51.70***
	(48.97–54.43)
6	53.59***
	(50.98–56.21)
7	55.49***
	(52.73–58.24)
8	57.38***
	(54.26–60.50)
9	59.27***
	(55.63–62.92)
10	61.16***
	(56.90–65.43)
11	63.06***
	(58.10–68.01)
12	64.95***
	(59.27–70.63)
CI in parentheses	

*** *p* < 0.01, ** *p* < 0.05, * *p* < 0.1

## Footnotes

1 Baseline questions regarding perceived level and satisfaction with level of involvement in treatment decisions, level, and satisfaction with level of GP and municipality involvement, and Health literacy. The Ten Item Personality Measure and questions regarding levels of importance concerning work, family, friend/acquaintances, leisure time, politics and religion were included in the first wave but are not applied in the current study

2 e-Boks is the most widely used secure digital mail platform in Denmark.
